# Restrictive prescription of antibiotics in preterm infants with premature rupture of membranes

**DOI:** 10.1186/s12887-022-03476-y

**Published:** 2022-07-12

**Authors:** Jakob Armann, Mario Rüdiger, Reinhard Berner, Lars Mense

**Affiliations:** 1 Department of Pediatrics, University Hospital Carl Gustav Carus Dresden, TU Dresden, Fetscherstrasse 74, 01307 Dresden, Germany; 2 Department of Pediatrics, Division of Neonatology & Pediatric Intensive Care Medicine, University Hospital Carl Gustav Carus Dresden, TU Dresden, Fetscherstrasse 74, 01307 Dresden, Germany

**Keywords:** Early onset sepsis, Antibiotic stewardship, PPROM

## Abstract

**Background:**

In preterm infants with premature rupture of membranes, antibiotic treatment is frequently started but rates of early onset sepsis are lower. In line with national guidelines, a stratified approach in the decision to start antibiotic treatment using maternal history, clinical impression and biomarkers has been implemented in our level III neonatal center and its results are evaluated.

**Methods:**

Retrospective cohort study of all preterm newborns with rupture of membranes at least 1 h prior to delivery admitted to our tertiary neonatal intensive care unit. Data on antibiotic exposure, mortality and major neonatal complications were extracted from the electronic patient charts to evaluate the effects and safety of our stratified approach.

**Results:**

Four hundred fifty-six infants met the inclusion criteria. 120 (26%) received primary antibiotics whereas 336 (74%) did not. Of those receiving primary antibiotics, 13 (11%) had a blood culture positive sepsis, 46 (38%) met the criteria of clinical sepsis and in 61 (51%) sepsis was ruled out and antibiotics were stopped after 48-96 h. All infants with blood culture positive sepsis were identified and treated within the first 24 h of life using this approach. None of the 336 infants who were not started on antibiotics primarily needed antibiotic therapy within the first 5 days of life. There were no deaths or major neonatal complications in the group that did not receive empiric antibiotics.

**Conclusions:**

Our stratified approach for preterm infants with premature rupture of membranes allows a safe reduction of antibiotic exposure even in this high risk population. As a result, only 25% of high risk preterm newborns are treated with antibiotics of which more than half receive less than 5 days of treatment. To treat one infant with blood culture positive sepsis, only 9 infants receive empiric antibiotics.

**Supplementary Information:**

The online version contains supplementary material available at 10.1186/s12887-022-03476-y.

## Background

Neonatal sepsis is a major cause of mortality and morbidity in infants [1]. Early onset sepsis (EOS) is caused predominantly by bacteria of the maternal recto-vaginal flora making premature rupture of membranes a major risk factor for EOS [2, 3]. Given the subtle, non-specific findings of neonatal sepsis paired with the high incidence of clinical instability in this age group, most preterm infants with preterm premature rupture of membranes (PPROM) are therefore started on empiric antibiotics and oftentimes treated for a prolonged period of time despite sterile blood cultures. This leads to a relevant overuse of antibiotics in this age group [4] with numbers needed to treat to potentially benefit one infant with neonatal sepsis of around 40, making antibiotics the most commonly used medication in neonatal intensive care units [5]. In addition to an increase in resistance, antibiotics can have well described side effects for the individual patient: Early antibiotic exposure is associated with short term effect as an increased risk of invasive candidiasis, necrotizing enterocolitis (NEC), late onset sepsis (LOS) and death [6] but also with allergic diseases, obesity, diabetes and inflammatory bowel disease later in life likely due to changes in the infants microbiome [7].

The use of biomarkers in combination with clinical assessment to support treatment discontinuation after 36-72 h in the setting of negative blood cultures is well described and recommended in national and international guidelines. However, their use to guide the decision to start antibiotics – especially in high-risk populations – is still controversial.

In our institution a combination of maternal risk factors, clinical presentation and biomarkers (WBC, I/T ratio, CrP and IL-6) is routinely used to guide the decision in regards of empiric antibiotics in newborns. This study aims to evaluate this approach for preterm infants with PPROM who are at high risk for EOS.

## Methods

### Study population and data acquisition

This retrospective study comprised all newborns of less than 37 weeks gestational age with rupture of membranes at least 1 h prior to delivery, born between January 2015 and March 2019 and admitted to the Division of Neonatology & Pediatric Intensive Care of the Department of Pediatrics at the University Hospital Carl Gustav Carus, Dresden, Germany. Babies born in referring hospitals were excluded. The Division of Neonatology & Pediatric Intensive Care is a level III neonatal center and includes a neonatal intensive care unit as well as an intermediate care and rooming-in unit. Electronic patient charts were reviewed retrospectively to collect clinical, laboratory and microbiological data. Microbiological and laboratory results as well as data on treatment with inotropes, vasopressors and blood products from the first 7 days of life were acquired. Death and major neonatal complications such as intraventricular hemorrhage grade 2 or higher (IVH), necrotizing enterocolitis (NEC) and bronchopulmonary dysplasia (BPD) were reviewed until discharge from hospital.

### Patient management

Newborns at risk of early onset sepsis are managed at our institution as follows: If clinically indicated at birth, a blood culture of at least 1 ml blood and a serum sample for C-reactive protein (CRP) and interleukin 6 (IL-6) analysis is drawn from the placenta after delivery, ensuring sterile management of the placenta until blood sampling. A complete blood cell count (CBC) is drawn during the insertion of a peripheral venous catheter. If sepsis evaluation is indicated after the delivery room management, blood culture and samples for laboratory analyses (including CBC, CRP and IL-6) are drawn from a peripheral poke with at least 0.5 ml blood for the blood culture.

Antibiotics are started as per the discretion of the attending neonatologist. The decision is based on maternal risk factors (PPROM, maternal fever, elevated WBC or CrP, vaginal purulent discharge), clinical presentation of the neonate (respiratory stability, blood pressure, heart rate, capillary refill time) and biomarkers (WBC, I/T ratio, CRP and IL-6). First line antibiotics for EOS are ampicillin and gentamicin. The microbiological results of antenatal vaginal swabs are incorporated into the decision and can reason second line antibiotics such as ampicillin and a cephalosporine of the cefotaxime group or treatment with a carbapenem.

Forty-eight hours after the initiation of antibiotic treatment, analysis of CBC and CRP is repeated. The decision to discontinue antibiotics is based on blood culture results, laboratory data and the clinical impression of the newborn.

### Statistics and definitions for this study

Four groups of patients were formed based on the blood culture results and their antibiotic therapy: “Blood culture positive sepsis (BCxS)”, “Clinical sepsis (CS)”, “Rule out sepsis” and “No antibiotic treatment”. Blood culture positive sepsis (BCxS) was defined as growth of any bacteria but coagulase negative staphylococci in a blood culture and antibiotic treatment for at least 5 days. Clinical sepsis (CS) was defined as antibiotic treatment for at least 5 days with sterile blood culture. Rule out sepsis was defined as antibiotic treatment of less than 5 days and a sterile blood culture.

We defined abnormal lab values on day of life (DOL) one as follows: White blood cell count (WBC) < 7 or > 25 GPT/L, I/T-ratio > 0.20, CRP > 10 mg/L or IL-6 > 1000 pg/mL.

We defined all normal lab values on DOL one as follows: WBC 7–25 GPt/L, I/T-ratio ≤ 0.20, CRP ≤ 10 mg/L and IL-6 < 150. No calculation was performed for the two lab characteristics if more than one of the four values was missing.

Bacteria in blood cultures were grouped into the following three groups: (i) True pathogens included *E. coli*, *H. influenzae*, *S. agalactiae*, *S. parasanguinis* and *S. mitis*. (ii) Likely contaminants included *C. imitans*, *M. luteus*, *Dermabacter hominis* and Sanguibacter spp. (iii) Coagulase negative staphylococci included *S. caprae*, *S. warneri*, *S. haemolyticus*, *S. hominis* and *S. epidermidis*.

Data was not normally distributed and is expressed as median (25th; 75th percentile) for metrical data and as frequency for categorical data. Statistical significance was tested using the Mann Whitney U test for metrical data and Fisher’s exact test for categorical data. Alpha was not adjusted for multiple testing. *p* < 0.05 was deemed statistically significant. Analyses were performed using IBM SPSS 25.0 and Microsoft Excel 2010. A sample size calculation was performed prior to the study with an expected rate of 5% newborns with a positive blood culture in the study population and the goal to identify 20 blood-culture positive preterm infants.

## Results

Data from 456 neonates were included in the study (Table 1). 120 (26%) neonates received primary antibiotics whereas 336 (74%) did not. Male newborns were overrepresented in the BCxS group (*p* = 0.04).

**Table 1:** Patient characteristics

	Blood culture positive sepsis	Clinical sepsis	Rule out sepsis	No antibiotics
N	13	46	61	336
Gestational age (weeks)	29 (27; 31.5)	30 (25.75; 32)	31 (28; 33)	34 (33; 35)
Birth weight (kg)	1.29 (0.99; 1.78)	1.45 (0.81; 1.93)	1.73 (1.28; 2.15)	2.3 (1.92; 2.59)
ROM (h)	24 (3; 96)	43 (3; 294)	96 (7.5; 264)	6 (3; 21.9)
Delivery per Caesarean section	8 (61.5%)	35 (76.1%)	45 (73.8%)	171 (50.9%)
APGAR 1 min	6 (5; 8)	6 (5; 7)	7 (5; 8)	8 (7; 9)
APGAR 5 min	8 (6.5; 9)	8 (6; 8)	8 (7; 8)	9 (8; 10)
APGAR 10 min	8 (7.5; 9)	8.5 (8; 9)	8 (8; 9)	9 (9; 10)
Gender (m:f)	5.5:1	1.7:1	1.4:1	1.1:1
Singleton (singleton: multiple)	12:1	8.2:1	6.6:1	2.1:1

Metrical data is presented as median (25th; 75th percentile)

### Laboratory data

Initial laboratory data was available in all newborns receiving antibiotic therapy. In 50 out of 336 newborns not receiving antibiotics during their first week of life, laboratory work-up was not performed. At birth, newborns with BCxS had lower WBC than all other groups and the I/T ratio was higher than in newborns not receiving antibiotics (Table 2). IL-6 at birth was not significantly different between BCxS and CS but higher than in rule out sepsis and no antibiotics. On follow up after 48 h of antibiotic treatment, CRP was higher in BCxS and CS than in rule out sepsis.

**Table 2:** Laboratory data per patient group

	Blood culture positive sepsis	Clinical sepsis	Rule out sepsis	No antibiotics
N (WBC on DOL 1)	13	46	61	286
WBC on DOL 1	8.4 (6.4; 14.2)	13.7 (11.6; 18.2)^**^	16.5 (11.9; 22.1)^**^	12.9 (10.6; 16.0)^**^
I/T ratio on DOL 1	0.17 (0.06; 0.47)	0.14 (0.05; 0.21)	0.09 (0.04; 0.13)	0.06 (0.02; 0.12)^* ##^
CRP on DOL 1	0.9 (0.3; 6.0)	0.3 (0.3; 1.1)^*^	0.3 (0.3; 0.3)^***^	0.3 (0.3; 0.3)^*** ###^
IL-6 on DOL 1	1651 (12; 13,795)	86 (9; 739)	21 (7; 106)^** ##^	6 (4; 12)^*** ###^
WBC at 48 hours	11.4 (7.3; 14.3)	12.1 (9.4; 15.3)	12.4 (9.7; 17.6)	
I/T at 48 hours	0.14 (0.09; 0.21)	0.08 (0.03; 0.19)	0.04 (0.02; 0.12)^**^	
CRP at 48 hours	10.2 (4.0; 36.1)	1.8 (0.6; 7.8)^**^	0.6 (0.3; 1.4)^*** ##^	

Metrical data is presented as median (25th; 75th percentile)

^*^p < 0.05, ^**^p < 0.01, ^***^p < 0.001 compared to blood culture positive sepsis

^#^*p* < 0.05, ^##^*p* < 0.01, ^###^*p* < 0.001 compared to clinical sepsis

### Mortality and morbidity

No deaths or major neonatal complications occurred in newborns not receiving antibiotics after birth (Table 3).

**Table 3:** Mortality and morbidity during hospital stay per patient group

	Blood culture positive sepsis	Clinical sepsis	Rule out sepsis	No antibiotics
Deaths	0 (0%)	3 (6.5%)	4 (6.6%)	0 (0%)^##^
IVH > grade 1	4 (31%)	5 (11%)	2 (3.3%)**	0 (0%)^*** ###^
NEC medical	0 (0%)	1 (2.2%)	0 (0%)	0 (0%)
NEC surgical	1 (7.7%)	0 (0%)	0 (0%)	0 (0%)^*^
BPD	0 (0%)	8 (17%)	3 (4.9%)	0 (0%)^###^

^*^p < 0.05, ^**^p < 0.01, ^***^p < 0.001 compared to blood culture positive sepsis

^##^*p* < 0.01, ^###^*p* < 0.001 compared to clinical sepsis

Seven neonates died during hospital stay (1.5% mortality, 6/7 born under 28 weeks gestational age): One newborn was born at 24 weeks with early-onset septic shock and multi-organ failure despite administration of antibiotics in the delivery room and full intensive care until care was redirected after 7 days. Two had rupture of membranes at 15 weeks of gestation and died of lung hypoplasia and pulmonary hypertension. The others had non-infectious congenital complications such as bilateral renal agenesis, congenital diaphragmatic hernia, early pulmonary emphysema and pulmonary hypertension. In all seven deceased newborns the initial blood culture was sterile but antibiotics started on the day of birth.

### Antibiotic exposure and decision making

Of those newborns receiving antibiotics 13 (11%) had BCxS, 46 (38%) a CS and in 61 (51%) sepsis was ruled out and antibiotics were stopped after 48–96 h. All infants with BCxS were identified in the first 24 h and started on antibiotics. None of the 336 infants who were not started on primary antibiotics needed secondary antibiotic therapy in the first 5 days of life.

Gestational age seems to be incorporated in the decision making: 89% of newborns ≤26 weeks were started on empiric antibiotics despite all lab values being normal, but only 4% of 35–36 week preterm newborns (Table 4). In higher gestational age groups, not all newborns with abnormal lab values were started on antibiotics (15% in 35–36 week preterm newborns) but all of those in the most immature group ≤26 weeks (Table 4).

**Table 4:** Antibiotic exposure and results of laboratory work-up and blood cultures, divided by gestational age groups

	≤ 26 weeks	27–31 weeks	32–34 weeks	35–36 weeks
N	26	83	192	155
Antibiotics on DOL 1 with abnormal labs	10/10 (100%)	24/28 (86%)	6/23 (26%)	2/13 (15%)
Antibiotics on DOL 1 despite all normal labs	8/9 (89%)	19/50 (38%)	19/153 (12%)	3/77 (4%)
Blood culture with a true pathogen	2/26 (8%)	7/79 (9%)	2/139 (1%)	1/34 (3%)
Blood culture with a likely contaminant	0/26 (0%)	1/79 (1%)	1/139 (1%)	2/34 (6%)
Blood culture with CONS	3/26 (12%)	3/79 (4%)	5/139 (4%)	0/34 (0%)
Blood culture sterile	21/26 (81%)	68/79 (86%)	131/139 (94%)	31/34 (91%)
No blood culture taken	0/26 (0%)	4/83 (5%)	53/192 (28%)	121/155 (77%)
Infants exposed to antibiotics	25/26 (96%)	49/83 (59%)	37/192 (19%)	8/155 (5%)
Number of infants exposed to antibiotics per blood culture with a true pathogen	12.5	7	18.5	8

The decision to stop antibiotics was also affected by gestational age in those newborns which were started on antibiotics on DOL 1 despite all normal lab values and did not have a positive blood culture after 48 h: 62.5% (5/8) of those newborns ≤26 weeks were treated with antibiotics for at least 5 days but only 30% (15/50) of those newborns above 26 weeks.

Nevertheless, only 9 premature infants were treated with antibiotics to treat one BCxS. Depending on the gestational age group, 7 to 18.5 newborns were exposed to antibiotics per positive blood culture with a true pathogen (Table 4).

### Blood culture results

Out of 278 blood cultures, truly pathogenic bacteria grew in 12, CONS in 11 and likely contaminants in 4. Of all lab values, only CRP was statistically significantly different between neonates with true pathogens and CONS in their blood culture (Supplement, Table 1).

The rate of positive blood culture results with true pathogenic bacteria was 8% in those newborns with 18–96 h PPROM but only 4 and 3% in those < 18 h and > 96 h PPROM respectively (*p* = 0.24).

## Discussion

Our study presents outcome data for preterm neonates with high risk for EOS after adopting an approach to eliminate the general use of empiric antibiotics and using a combination of maternal risk factors, clinical presentation and biomarkers instead.

Incorporating this strategy, we avoided antibiotic exposure after birth in the majority of preterm infants with PPROM. Most importantly this was achieved without any increase in infant mortality or morbidity. None of the 336 premature infants who were not treated died or developed major neonatal morbidities. Furthermore all infants with EOS – clinical or blood culture positive – were identified and treated within the first 24 h of life suggesting that our strategy in using biomarkers in the decision to start antibiotics for EOS did not lead to any delay in antibiotics in infants who needed them and making it therefore a safe approach.

Gestational age is one of the major risk factors for EOS [8] which is also reflected in our data showing a blood culture proven EOS rate of about 8% in infants < 32 weeks gestational age compared to a rate of < 1% in premature infants of 32 weeks gestational age and higher. Our approach allows for some clinical judgement of the neonatologist regarding initiation and discontinuation of antibiotics although having clearly defined criteria as lined out above. Unsurprisingly the antibiotic exposure in our cohort is significantly higher in lower gestational age infants reflecting the clinicians concern in trusting biomarkers in especially high-risk patients. Interestingly though, the EOS rate did not differ between infants below 26 and 27–31 weeks gestational age, whereas antibiotic exposure and especially antibiotic exposure despite normal biomarkers was significantly more often seen in the younger gestational age group. Given the lack of mortality and morbidity in the antibiotic free infants, our data suggests that an adoption of our biomarker strategy even in the youngest age group would most likely be safe and gestational age alone does not justify antibiotic treatment. It is concerning that antibiotics were not discontinued routinely in the most immature infants after 48–96 h despite having normal lab values on DOL 1 and a negative blood culture.

Data on PPROM and the risk of EOS is controversial. Some studies suggest that PPROM alone does not increase the risk of EOS [9, 10] while others do [11]. Nevertheless, since the American Academy of Pediatrics suggests empirical antibiotic therapy for infants with PPROM, antibiotic exposure in those newborns is common [12]. In our cohort especially infants with longer PPROM are treated with primary antibiotics. Given the doubled EOS rate in infants with PROM of 18–96 h compared to infants with PPROM less than 18 h this approach seems rational. Infants with PPROM of more than 96 h, however, have the same EOS rate as infants with short PPROM suggesting that very long PPROM is not an independent risk factor for EOS and that these infants do not necessarily need immediate antibiotic administration after birth.

Appropriate sample collection for blood cultures is of utmost importance in the identification of neonatal sepsis [13]. At least 1 ml blood should be collected to ensure appropriate sensitivity [14]. Incorporating biomarkers in the decision to start and discontinue antibiotics does not question the importance of blood cultures.

The retrospective study design has to be mentioned as a limitation. Generalisability of our results is high since our data reflect a long period of routine clinical care in the neonatal intensive care unit. Future prospective research to investigate associations between restrictive prescription of antibiotics and outcome is warranted.

## Conclusions

Strategies to safely prevent unnecessary antibiotic exposure in neonates are urgently needed, given the rising antibiotic resistance and the knowledge on short term side effects, toxicities as well as the growing awareness of lasting adverse effects to the microbiome. Our approach of laboratory evaluation, close clinical observation without routine antibiotics even in high risk infants might contribute to achieve this goal.

## Supplementary Information

**Additional file 1: Supplement Table 1.** Clinical charcteristics and laboratory data of patients with positive blood cultures, grouped by blood culture result.

## Data Availability

The datasets used and analysed during the current study are available from the corresponding author on reasonable request.

## Abbreviations

BCxS: Blood culture positive sepsis
BPD: Bronchopulmonary dysplasia
CBC: Complete blood cell count
CS: Clinical sepsis
EOS: Early onset sepsis
IVH: Intraventricular hemorrhage
LOS: Late onset sepsis
NEC: Necrotizing enterocolitis
PPROM: Preterm premature rupture of membranes
WBC: White blood cell count
