# Supplementary material for: Restrictive prescription of antibiotics in preterm infants with premature rupture of membranes
Source: BMC Pediatr. 2022 Jul 12;22:408. doi: 10.1186/s12887-022-03476-y (PMC9275236; doi:10.1186/s12887-022-03476-y)
Supplement: Supplementary file 1 — Additional file 1: Supplement Table 1. Clinical charcteristics and laboratory data of patients with positive blood cultures, grouped by blood culture result. [file 12887_2022_3476_MOESM1_ESM.docx]

**SUPPLEMENT**

Supplement Table 1. Clinical charcteristics and laboratory data of patients with positive blood cultures, grouped by blood culture result.

|  | True pathogen | Likely contaminant | CONS |
| --- | --- | --- | --- |
| N | 12 | 4 | 11 |
| Gestational age (wks) | 34 (32; 35) | 34 (30.75; 35.75) | 31 (26; 32) |
| Birth weight (kg) | 2.17 (1.74; 2.55) | 2.04 (1.45; 2.72) | 1.56 (0.74; 1.9) |
| ROM (h) | 24 (3.75; 96) | 23.5 (5; 765) | 18 (4; 120) |
| Delivery per Caesarean section | 8 (67%) | 0 (0%) | 10 (91%) |
| APGAR 1 min | 6.5 (5; 8) | 9 (6.75; 9) | 6 (4; 8) |
| APGAR 5 min | 8 (6.25; 9) | 8 (7.25; 8.75) | 7 (6; 8) |
| APGAR 10 min | 8.5 (7.25; 9) | 9 (8.25; 9) | 8 (8;9) |
| Gender (m:f) | 5:1 | 4:0 | 0.6:1 |
| Singleton (singleton : multiple) | 11:1 | 4:0 | 1.75:1 |
| WBC initially | 8.3 (6.2; 15.1) | 15.4 (10.1; 17.6) | 12.1 (9.2; 16.5) |
| I/T ratio initially | 0.17 (0.06; 0.47) | 0.02 (0.02; 0.02) | 0.09 (0.06; 0.25) |
| CRP initially | 1.7 (0.4; 6.1) | 0.3 (0.3; 0.3) | 0.3 (0.3; 0.8) * |
| IL-6 initially | 1795 (12; 13828) | 11 (5; 16) | 23 (5; 764) |
| WBC in follow up | 11.4 (8.2; 14.5) | 4.76 (4.75) | 13.8 (8.5; 26.4) |
| I/T in follow up | 0.14 (0.09; 0.21) | 0.13 | 0.22 (0.13; 0.27) |
| CRP in follow up | 10.1 (5.2; 36.5) | 0.6 (0.3) | 0.4 (0.3; 4.8) ** |

* p < 0.05 against True pathogens, ** p < 0.01. Likely contaminants were not tested for statistical significance due to the small sample size.
